# Supplementary material for: The Systems Biology Research Tool: evolvable open-source software
Source: BMC Syst Biol. 2008 Jun 29;2:55. doi: 10.1186/1752-0509-2-55 (PMC2446383; doi:10.1186/1752-0509-2-55)
Supplement: Additional file 1 — SBRT Archive. An archive of the current version of the Systems Biology Research Tool. [file 1752-0509-2-55-S1.zip › sbrt-1.4.0/doc/developers_guide/api/sbrt/kernel/fba/comp/CdHarFluxVectorComputer.Results.html]

CdHarFluxVectorComputer.Results


|  |  |  |
| --- | --- | --- |
| **Overview** \| **Package** \| **Class** \| **Use** \| **Tree** \| **Deprecated** \| **Index** \| **Help** | |  |
| **PREV CLASS**   **NEXT CLASS** | **FRAMES**    **NO FRAMES**     **All Classes** |  |
| SUMMARY: NESTED \| FIELD \| CONSTR \| METHOD | DETAIL: FIELD \| CONSTR \| METHOD |  |


---


## sbrt.kernel.fba.comp Class CdHarFluxVectorComputer.Results

```
java.lang.Object
  sbrt.kernel.fba.comp.CdHarFluxVectorComputer.Results
```

**Enclosing class:**: CdHarFluxVectorComputer

---

``` public class CdHarFluxVectorComputer.Results extends java.lang.Object ```

This class is used to represent the results of a coordinate-direction
hit-and-run flux vector computation.

**Author:**
:   This class was written and documented by Jeremiah Wright while in
    the Wagner lab.

---

| **Method Summary** | |
| --- | --- |
| `java.util.Map<java.lang.String,java.lang.Double>` | `getFluxVector()`             Returns the random interior flux vector that was computed. |
| `int` | `unchangedFreeVariables()`             Returns the number of free variables whose values are the same as they were in the initial flux vector. |

**Methods inherited from class java.lang.Object**

`clone, equals, finalize, getClass, hashCode, notify, notifyAll, toString, wait, wait, wait`

**Method Detail**

### getFluxVector

```
public java.util.Map<java.lang.String,java.lang.Double> getFluxVector()
```

:   Returns the random interior flux vector that was computed.

    :   **Returns:**: the random interior flux vector that was computed.

---


### unchangedFreeVariables

```
public int unchangedFreeVariables()
```

:   Returns the number of free variables whose values are the same as
    they were in the initial flux vector. Ideally, this number should
    equal 0.

    :   **Returns:**: the number of free variables whose values are the same as they were in the initial flux vector.


---


|  |  |  |
| --- | --- | --- |
| **Overview** \| **Package** \| **Class** \| **Use** \| **Tree** \| **Deprecated** \| **Index** \| **Help** | |  |
| **PREV CLASS**   **NEXT CLASS** | **FRAMES**    **NO FRAMES**     **All Classes** |  |
| SUMMARY: NESTED \| FIELD \| CONSTR \| METHOD | DETAIL: FIELD \| CONSTR \| METHOD |  |


---
